# Neonatal Quercetin Reduces Intestinal Oxidative Damage and Upregulates Tight Junction-Related Genes in a Mouse Experimental Model of Cerebral Palsy

**DOI:** 10.3390/antiox15040495

**Published:** 2026-04-16

**Authors:** Isla Ariadny Amaral de Souza Gonzaga Paz, Raul Manhães-de-Castro, Glayciele Leandro de Albuquerque, Osmar Henrique dos Santos Junior, Henrique José Cavalcanti Bezerra Gouveia, Nathalia Caroline de Oliveira Melo, Francisco Carlos Amanajás de Aguiar Junior, Ana Elisa Toscano

**Affiliations:** 1 Graduate Program in Neuropsychiatry and Behavioral Sciences, Center for Medical Sciences, Federal University of Pernambuco, Recife-Pernambuco 50670-901, Brazil; isla.ariadny@ufpe.br (I.A.A.d.S.G.P.); osmar.santosjunior@ufpe.br (O.H.d.S.J.); nathalia.melo@ufpe.br (N.C.d.O.M.); 2 Studies in Nutrition and Phenotypic Plasticity Unit, Center for Health Sciences, Federal University of Pernambuco, Recife-Pernambuco 50670-420, Brazil; raulmanhaesdecastro@gmail.com (R.M.-d.-C.); glaleandroalbuquerque@gmail.com (G.L.d.A.); 3 Department of Anatomy, Université du Québec à Trois-Rivières, 3351 Boulevard des Forges, Trois-Rivières, QC G9A 5H7, Canada; henrique.jose.cavalcanti.bezerra.gouveia@uqtr.ca; 4 Professional Master Program in Biology Teaching, Federal University of Pernambuco, Recife 55670-901, Brazil; francisco.amanajas@ufpe.br; 5 Nursing Unit, Vitória Academic Center, Federal University of Pernambuco, Vitória de Santo Antão-Pernambuco 55608-680, Brazil

**Keywords:** cerebral palsy, gut–brain axis, mucin, oxidative stress, quercetin, tight-junction proteins

## Abstract

Cerebral palsy (CP) is a non-progressive neurological condition associated with neuroinflammation, motor impairments, and gastrointestinal dysfunction mediated by the gut–brain axis. Preserving the intestinal epithelial barrier integrity may represent a therapeutic strategy, and quercetin is a bioactive compound with potential intestinal protective effects. This study investigated the effects of neonatal quercetin treatment on morphometric parameters, oxidative stress markers, and epithelial barrier gene expression in an experimental CP model. Wistar rats were distributed into four groups according to health status and treatment with a vehicle (V) or quercetin (Q, 10 mg/kg, intraperitoneally): healthy control (CV and CQ) and CP (CPV and CPQ) (n = 10/group). Intestinal morphology, oxidative stress markers, and gene expression (occludin, zonulin, and mucin 2) were evaluated. CP animals showed segment-specific alterations, with structural impairment predominantly in the ileum and increased oxidative damage in the jejunum. Quercetin attenuated oxidative stress markers and modulated antioxidant enzyme activity in CP, increased jejunal tight-junction gene expression in both healthy and CP groups, and enhanced MUC2 expression only in healthy animals, without fully reversing CP-induced morphological changes. In conclusion, neonatal quercetin modulates oxidative stress and epithelial barrier-related gene expression, supporting its potential as an adjuvant strategy for intestinal barrier protection in experimental CP.

## 1. Introduction

Cerebral palsy (CP) is a non-progressive clinical condition resulting from lesions in the immature brain. It is commonly associated with perinatal hypoxia/ischemia and encompasses a heterogeneous group of permanent disorders affecting motor and postural development [1,2]. Perinatal hypoxia associated with early motor restriction can compromise the bidirectional communication of the gut–brain axis [3], resulting in gastrointestinal alterations (abdominal distension, poor tolerance to diet and impaired speed of intestinal transit, among others) and exacerbating local and systemic inflammation [4,5]. This can also lead to increased lipid peroxidation, impaired tight junction-related gene expression, and structural changes in the intestinal epithelium [6]. Taken together, these findings suggest that improving the integrity of the intestinal barrier could be an effective way to mitigate the systemic dysfunction associated with neurological disorders such as CP [4].

In addition to its role in digestion and nutrient absorption, the intestine plays a central role in protecting the organism through its mucous barrier. This barrier mainly consists of a mucus layer rich in mucin, which is produced by goblet cells, as well as tight junctions, an integrated complex of structural proteins, such as occludin and zonulin, which regulate paracellular sealing between enterocytes [7]. The integrity of these components is essential for preserving the epithelium’s selective permeability, thereby preventing the translocation of pathogens, toxins, and pro-inflammatory metabolites into the systemic circulation [8]. However, when this barrier is compromised, a condition known as ‘leaky gut’ develops. This is characterized by increased intestinal permeability, chronic inflammation, oxidative stress, and systemic dysfunctions that are observed in several neurological conditions [9]. This highlights the need for adjuvant therapeutic strategies to mitigate these complications.

In this sense, bioactive plant-derived compounds, such as quercetin, have aroused interest thanks to their antioxidant, anti-inflammatory and gene-modulating properties [10]. A flavonoid widely found in fruits, vegetables and grains, quercetin can activate the AMPK and Nrf2 pathways and inhibit NF-κB, microglia-derived oxidative stress and TLR4-mediated inflammation, thereby reducing the production of pro-inflammatory cytokines [11,12]. This suggests its potential to regulate neuroinflammation, even in complex pathophysiological conditions, such as neonatal brain injury [13]. To date, no study has investigated the modulation of the intestinal barrier by quercetin in experimental CP. Therefore, we aimed to investigate the effects of neonatal quercetin treatment in an experimental model of cerebral palsy by evaluating, in the intestine, (i) morphometric parameters, (ii) oxidative stress markers, and (iii) epithelial barrier gene expression (occludin, zonulin and mucin 2) to determine whether quercetin can preserve gut integrity and modulate oxidative damage, providing insights into the involvement of the gut–brain axis.

## 2. Materials and Methods

### 2.1. Animals

The experimental protocol described below adhered to the standards of the National Council for Animal Control and Experimentation and the National Institutes of Health Guide for the Care and Use of Laboratory Animals, as well as the recommendations of the ARRIVE guidelines (3R principles). It was also approved by the Committee on Ethics in the Use of Animals at the Federal University of Pernambuco/Brazil (n° 0078/2022).

Healthy male and female progenitors of rats of the Wistar lineage (Rattus norvegicus albinus) between 90 and 100 days of life, with weights ranging from 330 to 220 g, respectively, were obtained at the Department of Nutrition of the Federal University of Pernambuco and housed in polypropylene cages (46 × 34 × 20 cm) in a controlled environment (a 22 ± 2 °C temperature, an inverted light/dark cycle [lights on from 20:00 to 08:00 and lights off from 08:00 to 20:00], free access to water and standard feed for rodents [Nuvilab^®^, containing 29% protein, 60% carbohydrates (fiber: 8 g/100 g), 11% lipids and 3.47 kcal/g]) at the Unit for Studies in Nutrition and Phenotypic Plasticity/Federal University of Pernambuco, Brazil, during the entire experimental period.

To obtain the offspring, the animals were mated at a ratio of one male to two females, and pregnancy was confirmed by monitoring maternal weight gain. At birth, the litter size was standardized to eight pups per dam (±5–8 g/each), with a ratio of four males to four females. Mothers and their respective litters were randomly assigned to the experimental conditions (control or cerebral palsy). Due to the characteristics of the cerebral palsy model, all pups from the same litter were maintained under the same conditions to ensure adequate maternal care. Within each litter (n = 4 males and 4 females), male pups were subdivided into treatment groups, with 2 males receiving either quercetin or 2 males receiving the vehicle solution. Thus, the following groups were formed: Control + Vehicle (CV), Control + Quercetin (CQ), Cerebral Palsy + Vehicle (CPV), and Cerebral Palsy + Quercetin (CPQ), with 10 male pups per group.

#### 2.1.1. Experimental Model of Cerebral Palsy

The experimental model of CP was based on the protocols described by Strata et al. (2004) [14], Coq et al. (2008) [15] and Silva et al. (2016) [16]. The model associates perinatal anoxia with sensory-motor restriction of the hind limbs, simulating immobility conditions typical of CP. On the first (P1) and second (P2) postnatal days, the pups of the CPV and CPQ groups were placed in a glass chamber partially immersed in water at 37 °C and exposed to a continuous flow of nitrogen (100%) at 9 L/min for 12 min. After the procedure, the pups were kept at room temperature until the recovery of mucosal coloration and normalized breathing, being then returned to their mothers. Between P2 and P28, sensorimotor restriction was applied to the hind limbs with the use of an epoxy mold and adhesive tape for 16 h/day (20–12 h), which allowed only limited movements of the hip, keeping the limbs extended, without impairing maternal care or excretion of urine and feces [16].

#### 2.1.2. Neonatal Treatment with Quercetin

The animals, from P2 to P22, received quercetin (Sigma-Aldrich–Q4951, St. Louis, MO, USA) (CQ/CPQ, 10 mg/kg/day) or a vehicle solution (CV/CPV), administered intraperitoneally [13]. Quercetin was dissolved in ultra-pure dimethyl sulfoxide (DMSO) (Amresco–WN182–10 mL, Kensington, MD, USA) and further diluted in saline solution (1:9, *v*/*v*) to obtain a final concentration of 10 mg/mL. The final solution was administered at a volume of 1 µL/g body weight. The vehicle solution consisted of the same DMSO–saline ratio used for quercetin dilution.

In P22, the animals were weaned, and the dams and females were excluded from this study. The litters were kept together in the cage (4 males per dam) and accompanied until P33 (with drinking water and a standard diet of Nuvilab^®^ ad libitum), at which point represented an early post-weaning stage, enabling the evaluation of the sustained effects of quercetin administration after the neonatal period. On this same day, the animals were euthanized by decapitation, and portions of the jejunum and ileum were removed. The samples were stored in two forms: (i) some were fixed in 10% buffered formalin for 48 h, dehydrated in an increasing series of ethanol, clarified in xylene and embedded in paraffin wax for evaluation of histomorphometric characteristics; (ii) the remainder were stored at −80 °C for subsequent evaluation of enzyme activity and target gene expression.

### 2.2. Histomorphometric Analysis of the Small Intestine

Histomorphometric analysis was performed using a subset of animals (n = 7 per group), and semi-seriated (5 μm) cuts of the jejunum and ileum were stained with hematoxylin and eosin (HE). The slides were digitized using a Moticam 2300 (Motic, Loveland, OH, USA) with objectives of 10 and 40 and analyzed using the ImageJ^®^ v1.8.0_172 software (NIH, Bethesda, MD, USA). The villi length and width in their different regions (apex, middle and base), as well as the thicknesses of the mucosa, submucosa and muscular tunics, were evaluated.

### 2.3. Evaluation of Oxidative Stress Biomarkers in the Small Intestine

A subset of 5 animals per group was considered for all the oxidative stress analyses described below.

#### 2.3.1. Lipid Peroxidation

Malonaldehyde (MDA) was quantified through a reaction with thiobarbituric acid (TBA), as described by Buege and Aust (1978) [17]. The resulting chromophore was extracted with n-butanol, and its absorbance was measured at 535 nm. The results were expressed in nmol of TBARS/mg of protein.

#### 2.3.2. Protein Oxidation

Carbonyl protein was evaluated using the method described by Levine et al. (1990) [18]. The carbonyl groups reacted with 2,4-dinitrophenylhydrazine (DNPH), and the absorbance was measured at 370 nm. The results were expressed as nmol of carbonyls/mg of protein.

### 2.4. Evaluation of Antioxidant Enzyme Activity in the Small Intestine

Superoxide dismutase (SOD) activity was evaluated using the adrenaline auto-oxidation rate at 480 nm, expressed in U/mg of protein [19]. Catalase (CAT) activity was analyzed by measuring the decomposition of hydrogen peroxide (H_2_O_2_) at 240 nm, with the results expressed in U/mg of protein [20]. Glutathione S-transferase (GST) was quantified using the method proposed by Habig and Jakoby (1981) [21], which involved monitoring CDNB-GSH conjugate formation at 340 nm and expressing the results in U/mg of protein.

### 2.5. Evaluation of Gene Expression of Proteins Related to the Intestinal Barrier in the Jejunum

The gene expression of markers of intestinal barrier integrity was quantified by RT-qPCR using a subset of animals (n = 5/group). Total RNA was extracted from jejunum samples and converted to cDNA by reverse transcription. The genes of interest were amplified using specific primers (Table 1), and the data were normalized using the constitutive gene EEF1A1 as a reference [22,23].

### 2.6. Statistical Analysis

Data analysis was performed using the GraphPad Prism 8.0 software (GraphPad Software, Inc., La Jolla, CA, USA). The results were verified for normality of distribution using the Kolmogorov–Smirnov test. Parametric variables were analyzed using one- or two-way ANOVA, followed by Tukey’s post hoc test, while non-parametric variables were analyzed using the Kruskal–Wallis test and Dunn’s post hoc test. The significance level was set at 5% (*p* < 0.05). The results are expressed as means ± standard error of the means (SEM).

## 3. Results

### 3.1. Histomorphometric Analysis of the Small Intestine

#### 3.1.1. Jejunum

**Figure 1:**
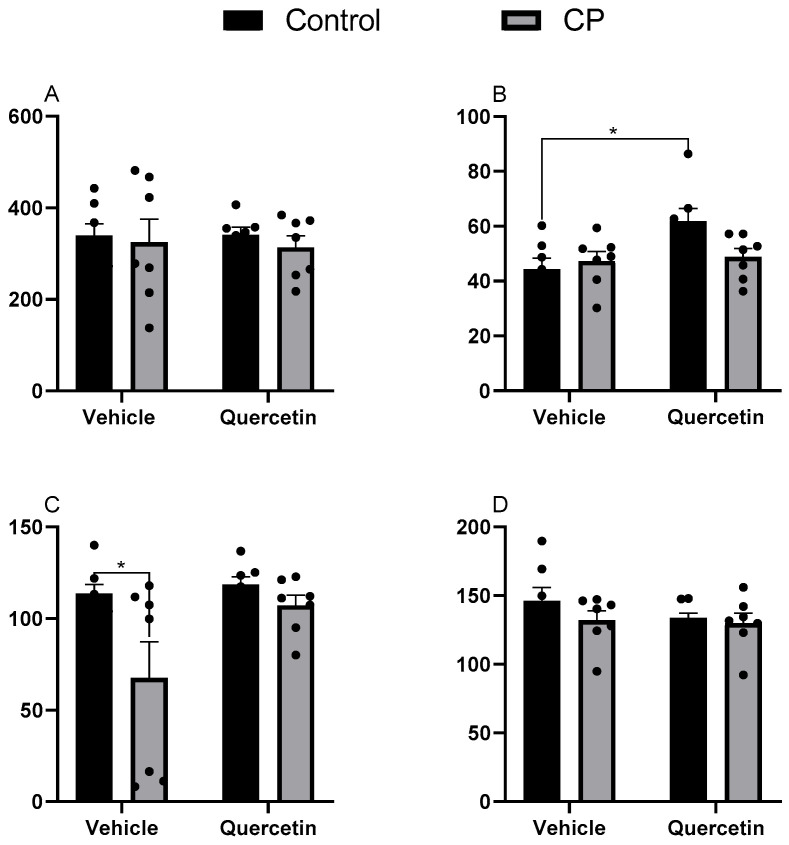
Morphometric parameters of jejunal villi at P33 in healthy animals or animals with CP, receiving or not receiving treatment with quercetin (10 mg/kg) intraperitoneally, from P2 to P22. CP: cerebral palsy. (**A**) Height (n = 7), (**B**) apex width (n = 7), (**C**) mid-width (n = 7), and (**D**) base width (n = 7). Semi-seriated sections (5 µm) were stained with hematoxylin and eosin (H&E). Values expressed as means ± standard error of the means with individual data points shown. Two-way ANOVA followed by Tukey’s post hoc test. * *p* < 0.05.

In this region, there were no main effects of cerebral palsy (F(1.24) = 0.43; *p* = 0.51), treatment with quercetin (F(1.24) = 0.02; *p* = 0.88) or the interaction between factors (F(1.24) = 0.03; *p* = 0.84) on the length of the villi (Figure 1A). When analyzing the width of the villi, distinct effects were identified according to the region observed. At the apex, there were no main effects of cerebral palsy [F(1.24) = 1.77; *p* = 0.19] or quercetin [F(1.24) = 0.32; *p* = 0.57], or any significant interactions between the factors. However, the post hoc test revealed that healthy animals treated with quercetin (CQ) exhibited wider apexes than healthy untreated controls (CV) (Tukey: *p* < 0.05), suggesting an independent effect of the compound on this group (Figure 1B).

In the median region, cerebral palsy significantly reduced villus width (F(1.24) = 7.07; *p* = 0.01), with no effect of quercetin (F(1.24) = 4.23; *p* = 0.05) or their interaction (F(1.24) = 2.58; *p* = 0.12) (Figure 1C). When evaluating the width of the base, no main effects were observed for paralysis [F(1.24) = 1.51; *p* = 0.23], quercetin [F(1.24) = 0.98; *p* = 0.33], or their interaction [F(1.24) = 0.49; *p* = 0.48] (Figure 1D). A similar result was observed for the thickness of the mucous membrane, where there was no effect of paralysis (F(1.24) = 1.78; *p* = 0.19), quercetin (F(1.24) = 0.77; *p* = 0.38), or their interaction (F(1.24) = 1.34; *p* = 0.25). Conversely, submucosal tunic thickness was significantly reduced in CPQ compared with CQ [F(1.24) = 9.05; *p* < 0.00], although no isolated effect of quercetin in healthy animals was identified [CQ vs. CV: F(1.24) = 0.23; *p* = 0.63], nor in the interaction between factors in the paralyzed state [CPQ vs. PC: F(1.24) = 1.06; *p* = 0.31]. The morphological characteristics of the jejunum are shown in Supplementary Figure S1.

#### 3.1.2. Ileum

**Figure 2:**
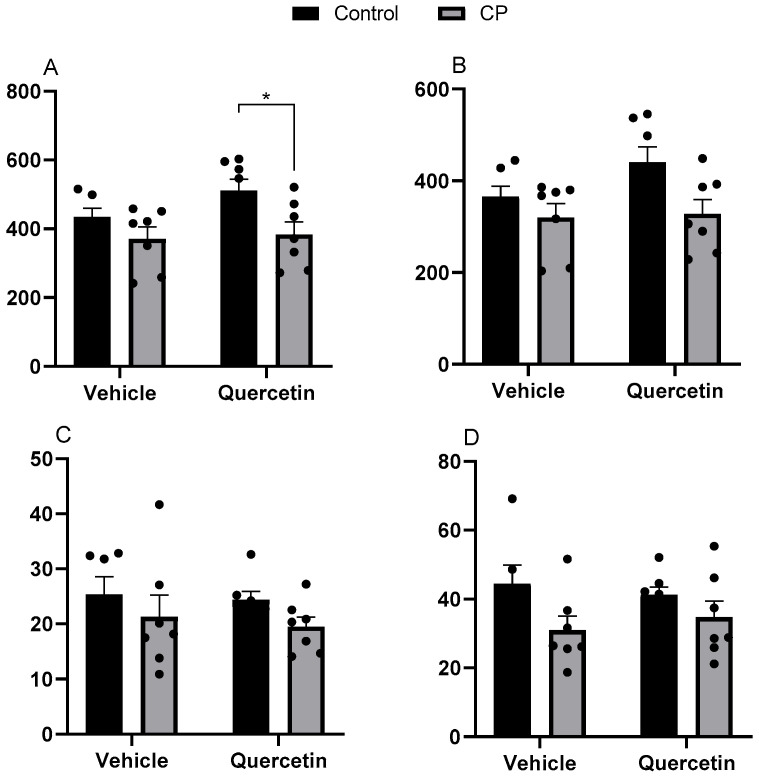
Morphometric parameters of ileal villi at P33 in healthy animals or animals with CP, receiving or not receiving treatment with quercetin (10 mg/kg), intraperitoneally, from P2 to P22 (n = 7). CP: cerebral palsy. (**A**) Height (n = 7), (**B**) apex width (n = 7), (**C**) mid-width (n = 7), and (**D**) base width (n = 7). Semi-seriated sections (5 µm) were stained with hematoxylin and eosin (H&E). Values are expressed as means ± standard error of the means with individual data points shown. Two-way ANOVA followed by Tukey’s post hoc test. * *p* < 0.05.

In the ileum, cerebral palsy resulted in a significant reduction in villus length, particularly in the CPQ group (F(1.24) = 23.50; *p* < 0.00), suggesting that quercetin was unable to prevent this damage in animals subjected to neonatal injury. Conversely, among healthy animals, quercetin promoted a slight increase in villous length (Figure 2A).

No significant effects were observed for the width of the villi apex for paralysis (F(1.24) = 2.56; *p* = 0.12), quercetin (F(1.24) = 0.00; *p* = 0.96), or the interaction between the two factors (F(1.24) = 2.27; *p* = 0.14) (Figure 2B). When evaluating the width of the middle region, a reduction was observed in animals with paralysis (F(1.24) = 9.21; *p* < 0.01), with no effect of quercetin (F(1.24) = 0.60; *p* = 0.44) or their interaction (F(1.24) = 0.58; *p* = 0.45) (Figure 2C). This was similar to the reduction observed in the base width of villi, which was also lower in animals with paralysis (F(1.24) = 14.02; *p* < 0.00), with no influence of quercetin (F(1.24) = 2.10; *p* = 0.15) or the interaction between the factors (F(1.24) = 1.02; *p* = 0.32) (Figure 2D).

As for the tunics, a reduction in the mucosal layer thickness was observed in paralyzed animals [F(1.23) = 6.83; *p* = 0.01], with no effect of quercetin [F(1.23) = 1.89; *p* = 0.18] or the interaction between factors [F(1.23) = 1.23; *p* = 0.27]. There were no differences in submucosal thickness between the groups [paralysis: F(1.23) = 2.63, *p* = 0.11; quercetin: F(1.23) = 0.25, *p* = 0.61; interaction: F(1.23) = 0.02, *p* = 0.88]. However, evaluation of the muscular tunica revealed that the paralysis condition was reflected in a reduction in the thickness of this layer [F(1.23) = 5.79; *p* = 0.02], with no influence from quercetin [F(1.23) = 0.00; *p* = 0.95] or the interaction between factors [F(1.23) = 0.72; *p* = 0.40]. The morphological characteristics of the ileum are shown in Supplementary Figure S2.

### 3.2. Analysis of Oxidative Stress in the Small Intestine

#### 3.2.1. Jejunum

**Figure 3:**
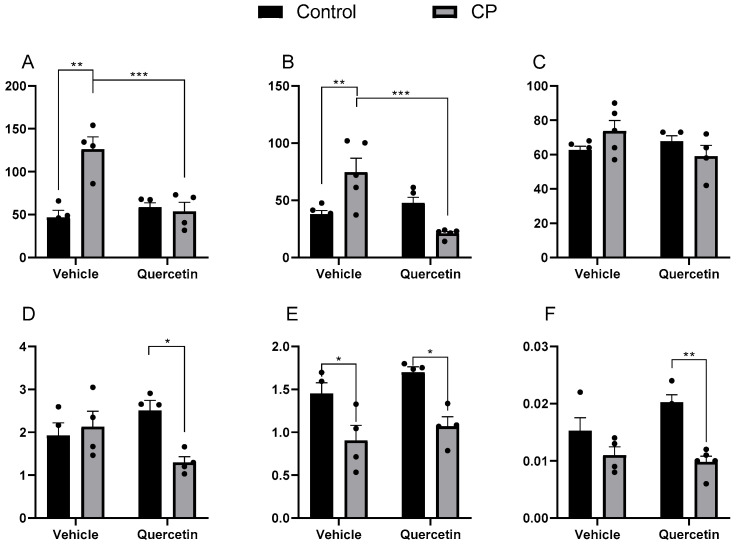
Markers of oxidative stress in the jejunal villi at P33 in healthy animals or animals with CP, receiving or not receiving treatment with quercetin (10 mg/kg), intraperitoneally, from P2 to P22. CP: cerebral palsy. (**A**) MDA (TBARS/mg of protein; n = 5), (**B**) carbonyls (nmol of carbonyls/mg of protein; n = 5), (**C**) SOD (U/mg of protein; n = 5), (**D**) CAT (U/mg of protein; n = 5), (**E**) GST (U/mg of protein; n = 5), and (**F**) total thiols (nmol/mg of protein; n = 5). Values expressed as means ± standard error of the means with individual data points shown. Two-way ANOVA followed by Tukey’s post-hoc test. * *p* < 0.05; ** *p* < 0.01; and *** *p* < 0.001.

There was a significant increase in MDA concentrations in CPV animals compared with CV controls [F(1.12) = 13.66; *p* < 0.001]. However, administering quercetin mitigated this increase in CPQ animals (CP vs. CPQ: F(1.12) = 8.95; *p* = 0.01), suggesting a significant interaction between paralysis and treatment (F(1.12) = 17.34; *p* < 0.00) (Figure 3A). When quantifying the carbonyls, there was no main effect of paralysis (F(1.16) = 0.52; *p* = 0.47); however, there was a significant effect of quercetin (F(1.16) = 0.22; *p* < 0.01), as well as of the interaction between the factors (F(1.16) = 21.47; *p* < 0.00) (Figure 3B). These findings suggest that quercetin plays an important role in modulating the reduction in protein oxidation in CP animals.

SOD activity was not influenced by paralysis (F(1.14) = 0.05; *p* = 0.82), quercetin (F(1.14) = 1.05; *p* = 0.32), or the interaction between the two factors (F(1.14) = 4.22; *p* = 0.05) (Figure 3C). Similarly, there was no main effect of paralysis (F(1.12) = 3.49; *p* = 0.09) or quercetin (F(1.12) = 0.22; *p* = 0.65) on CAT activity. However, a significant interaction between the factors was identified (F(1.12) = 6.99; *p* = 0.02), indicating differential modulation of this enzyme according to the animals’ pathophysiological state (Figure 3D).

GST activity was significantly reduced in individuals with paralysis (F(1.12) = 22.06; *p* < 0.00), with no effect observed for quercetin (F(1.12) = 2.67; *p* = 0.13) or the interaction between factors (F(1.12) = 0.11; *p* = 0.74) (Figure 3E). Finally, total thiol levels were reduced in paralyzed animals [F(1.13) = 22.69; *p* < 0.00], and although no effect of quercetin was observed [F(1.13) = 1.52; *p* = 0.24], an effect of the interaction between factors was observed [F(1.13) = 4.04; *p* < 0.01] (Figure 3F).

#### 3.2.2. Ileum

**Figure 4:**
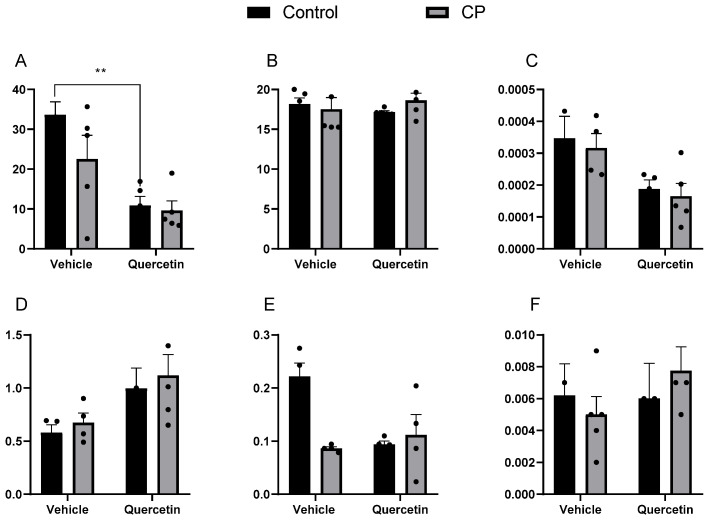
Markers of oxidative stress in the ileum at P33 in healthy animals or animals with CP, receiving or not receiving treatment with quercetin (10 mg/kg), intraperitoneally, from P2 to P22. CP: cerebral palsy. (**A**) MDA (TBARS/mg of protein; n = 5), (**B**) carbonyls (nmol of carbonyls/mg of protein; n = 5), (**C**) SOD (U/mg of protein; n = 5), (**D**) CAT (U/mg of protein; n = 5), (**E**) GST (U/mg of protein; n = 5), and (**F**) total thiols (nmol/mg of protein; n = 5). Values expressed as means ± standard error of the means with individual data points shown. Two-way ANOVA followed by Tukey’s post hoc test. ** *p* < 0.01.

MDA concentrations were not influenced by the cerebral palsy condition (F(1.16) = 2.68; *p* = 0.12) but showed a significant reduction in response to quercetin treatment in healthy animals (F(1.16) = 22.18; *p* < 0.00). There was also no significant interaction between the factors (F(1.16) = 1.69; *p* = 0.21) (Figure 4A). No significant effects of paralysis [F(1.16) = 0.18; *p* = 0.68], quercetin [F(1.16) = 0.00; *p* = 0.97], or the interaction between factors [F(1.16) = 1.26; *p* = 0.28] were observed for the carbonyls (Figure 4B).

The activity of SOD was not influenced by paralysis (F(1.14) = 0.27; *p* = 0.61), quercetin administration (F(1.14) = 9.28; *p* > 0.05), or the interaction between these factors (F(1.14) = 0.00; *p* = 0.94) (Figure 4C). Similarly, the effect of paralysis (F(1.14) = 0.43; *p* = 0.52), quercetin administration (F(1.14) = 6.87; *p* > 0.05), and the interaction between these factors (F(1.14) = 0.00; *p* = 0.94) were not significant for CAT (Figure 4D). Conversely, GST activity was influenced by paralysis [F(1.12) = 6.47; *p* = 0.03] and quercetin [F(1.12) = 4.89; *p* = 0.04], as well as a significant interaction between the two factors [F(1.12) = 11.00; *p* < 0.00] (Figure 4E). Finally, total thiol levels were unaffected by paralysis [F(1.15) = 0.02; *p* = 0.88], quercetin [F(1.15) = 0.50; *p* = 0.49], or the interaction between factors [F(1.15) = 0.67; *p* = 0.43] (Figure 4F).

### 3.3. Gene Expression of Occludin, Zonulin and MUC2 in the Jejunum

**Figure 5:**
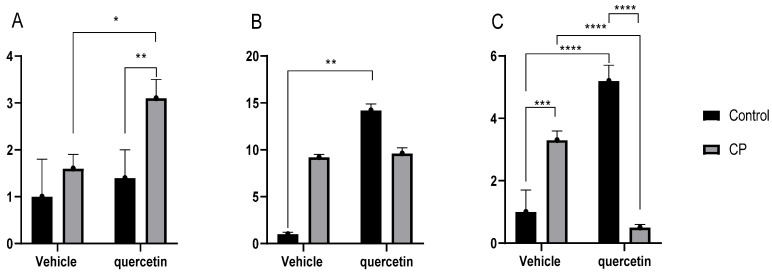
Gene expression of proteins in the jejunum at P33 in healthy animals or animals with CP, assessed by RT-qPCR (the EE1FA1 gene was used as the housekeeping control). Neonates received daily treatment with quercetin (10 mg/kg) or a vehicle, intraperitoneally, from P2 to P22. CP: cerebral palsy. (**A**) Occludin, (**B**) zonulin, and (**C**) MUC2 (n = 5/group). Data are presented as means ± SEM and were evaluated by two-way ANOVA followed by Tukey’s post hoc test. * *p* < 0.05; ** *p* < 0.01; *** *p* < 0.001; and **** *p* < 0.0001.

The analysis of gene expression of junction proteins and epithelial barrier components revealed relevant alterations associated with both cerebral palsy and quercetin treatment. For occludin, the main effect of paralysis was found [F (1,13) = 45,69; *p* < 0.001], with higher expression in CPV animals compared with CV animals. There was also the main effect of quercetin, which was reflected in a significant increase in expression in CQ animals in relation to CVs. In addition, there was a significant interaction between paralysis and quercetin [F (1.13) = 577.6; *p* < 0.00], indicating modulation of gene expression dependent on the physiopathological state of the animals (Figure 5A).

No main effect of paralysis was observed for zonulin [F(1,11) = 28.81; *p* > 0.05]. However, an increase in its expression was observed in CPQ animals compared with CPV animals, indicating a significant effect of quercetin [F(1,11) = 9.80; *p* < 0.00]. Additionally, a strong interaction was observed between the factors [F(1,11) = 245.1; *p* < 0.00], once again indicating that the modulation induced by quercetin depends on the animals’ pathophysiological condition (Figure 5B).

In relation to MUC2, there was no main effect of paralysis (F(1,13) = 17.61; *p* > 0.05), but a significant effect of quercetin was observed in healthy animals (F(1,13) = 12.02; *p* < 0.00), characterized by increased expression in the CQ group compared with the CV group. In contrast, no interaction between the factors was observed [F(1,13) = 4.03; *p* = 0.06] (Figure 5C).

## 4. Discussion

This study investigated the responses to neonatal quercetin treatment of histomorphometric parameters, oxidative stress markers, and the expression of key proteins of the intestinal epithelial barrier for the first time in an experimental cerebral palsy model. Brain injury can trigger persistent systemic inflammatory responses that bypass the central nervous system and reach peripheral tissues, such as the gastrointestinal tract [24].

In general, the findings demonstrate that perinatal neural damage compromises intestinal integrity by affecting the morphology, redox balance, and components of the mucosal barrier and paracellular connection. The administration of quercetin during the postpartum period following birth partially attenuates these effects, seemingly due to its antioxidant and anti-inflammatory actions, which can modulate the epithelial barrier [23], although they do not fully restore the structural parameters.

From a morphological standpoint, different repercussions of CP and quercetin were observed in the jejunum and ileum. More prominent structural damage was evident in the ileal portion of animals with CP, particularly in terms of villi length, middle region and base width, and mucous membrane and muscle thickness. These changes highlight the importance of considering segment-dependent intestinal responses when investigating the effects of experimental CP, and this may be due to regional differences in cell composition, immunological density, and epithelial plasticity [25].

The effects of quercetin on intestinal morphology depended on the intestinal segment and the pathophysiological status of the animals. In homeostatic conditions, particularly in the jejunum, quercetin modulated the villi architecture, increasing the width of their apices and, thus, the contact surface with nutrients, like that observed by Barrenetxe et al. (2006) [26]. In contrast, in the ileum, the effects of quercetin were less pronounced, which can be attributed to its emphasis on intestinal immunity and final absorption of specific nutrients such as bile salts and vitamin B12, which confers to this tissue lower adaptive morphological plasticity under nutritional or antioxidant stimuli such as quercetin, with responses more focused on immune modulation and less on structural remodeling [27].

In contrast, in animals with CP, the systemic inflammatory environment and oxidative stress resulting from perinatal neural injury may have restricted the beneficial effects of quercetin, causing it to act on inflammation rather than structural modulation. These findings corroborate those of Sukhotnik et al. (2018) [28] and Zou et al. (2016) [29], who demonstrated that quercetin could modulate intestinal morphological parameters in different health conditions and animal models, respectively. This has been associated with quercetin’s ability to (i) stimulate p-ERK, a key signaling protein that promotes cell proliferation and differentiation [30]; (ii) reduce caspase-3, thereby reducing cellular apoptosis [31]; (iii) preserve Nrf2 nuclear translocation, thereby supporting endogenous antioxidant responses [30]; and (iv) modulate the NF-κB pathway, thereby reducing inflammatory cytokine expression [32].

In CP, the activation of microglia promotes the sustained release of pro-inflammatory cytokines, such as TNF-α, IL-6, and IL-1β, which play a central role in amplifying and maintaining neuroinflammation [33]. These cytokines can reach the intestine via the systemic circulation, and, upon reaching the intestinal epithelium, these cytokines negatively modulate the integrity of the barrier by activating pathways such as NF-κB and MAPKs, which results in the intensification of intestinal oxidative stress [34], the disorganization of tight junctions, and an increase in paracellular permeability [35]. In this context, the findings of the present study reinforce the notion that the intestine has its physiology directly impaired by neonatal neuroinflammation associated with CP.

When analyzing intestinal oxidative stress, the effects of paralysis and quercetin were also distinct in the regions examined. The jejunum showed a greater negative impact of CP, with CPV animals presenting a redox imbalance, characterized by increased lipid oxidation products (MDA), reduced total thiols and decreased GST enzymatic activity, compared with CV. However, these repercussions were not observed in the ileum, contrary to what was reported by Liu et al. (2025) [36], who found that neonatal inflammatory lesions promoted systemic oxidative and inflammatory intestinal damage. This discrepancy may be attributed to the CP model used in the experiments, suggesting that oxidative repercussions appear to be segment-specific along the intestine in this condition. This may reflect regional differences in metabolic rate, cellular composition, and basal antioxidant capacity between the jejunum and ileum [25,27]. Therefore, the jejunum may be more susceptible to a redox imbalance induced by CP due to its higher absorptive activity and exposure to pro-oxidant substrates. In contrast, other intestinal segments, such as the ileum, may exhibit more efficient compensatory mechanisms.

The administration of quercetin modulated some of these effects in animals with CP (CPQ). In the jejunum, quercetin reduced the concentrations of MDA and carbonyl, thereby reducing lipid and protein oxidation. It also increased the antioxidant activity of GST and CAT. This suggests that quercetin acts as a modulator of the intestinal antioxidant response. It does this by stimulating enzymatic defense mechanisms and limiting the accumulation of oxidation products, and this is particularly important in animals that are more vulnerable to oxidative stress [23]. Interestingly, its repercussions in the ileum were only observed in healthy animals (CQ), where it reduced MDA concentrations and increased GST activity without influencing the other analyzed parameters, possibly reflecting a lower demand for antioxidant modulation in this segment. These results suggest that the antioxidant effects of quercetin depend on the intestinal segment and the physiological state of the host, having a greater impact in regions and conditions characterized by a pre-existing redox imbalance.

The analysis of intestinal barrier components revealed that occludin and zonulin expression was influenced by experimental conditions. Tight junctions are highly dynamic structures that undergo continuous remodeling in response to stress and inflammation, reflecting adaptive attempts to restore epithelial homeostasis. This characteristic was remarkable in the animals of CPV, which showed an increase in occludin expression compared with CV. This finding seems to reflect a compensatory response to intestinal stress and inflammation associated with an injury model, as already described in the literature [37], where the increase in expression of this component does not necessarily correspond to an improvement in barrier integrity, but to an attempt to attenuate and/or restore epithelial homeostasis in adverse conditions such as CP.

In CQ and CPQ animals, an increase in the expression of these components was observed, demonstrating the role of quercetin in modulating the connection between enterocytes and in regulating intestinal permeability, both in homeostatic and pathological conditions. This was similar to the effects observed in other animal models of intestinal inflammation treated with quercetin [11,38], and, mechanistically, these effects are associated with the inhibition of PKCδ signaling and consequent stabilization of protein complexes of the tight junction in intestinal epithelial models, culminating in increased transepithelial resistance and a reduction in paracellular permeability [39].

However, when its repercussions on MUC2 were evaluated, this was only observed in healthy animals (CQ vs. CV). Under these conditions, quercetin can stimulate pathways associated with the differentiation of goblet cells via modulation of the PKCα/ERK1-2 pathway, favoring increased mucin expression and secretory activity [40]. Conversely, under pathophysiological conditions (such as CP), the epithelium prioritizes cell proliferation and repair. This may reduce the energy and molecular resources allocated for secretory differentiation, limiting the response to modulation of MUC2 expression in CPQ animals.

The role of quercetin in modulating different inflammatory pathways is well-established: (i) activation of Nrf2, increasing HO-1, NQO1 and glutathione-linked enzymes [41]; (ii) inhibition of NF-κB, reducing pro-inflammatory cytokines [42]; (iii) regulation of MLCK and RhoA/ROCK, preserving the actin cytoskeleton, which is necessary for the maintenance of zonulin and occludin [43]; and (iv) activation of PI3K/Akt, favoring goblet cell differentiation and increasing MUC2 [40]. However, its benefits may vary depending on the pathophysiological conditions, age, administration method and the genetic characteristics of the species studied. These findings contribute to novel evidence regarding intestinal involvement in experimental CP.

Although exploring the interaction between quercetin and intestinal integrity in the context of experimental CP is unprecedented, some limitations should be mentioned: (a) The use of a single sex (males), a specific intervention window (P1–P21) and the way in which quercetin is administered (IP) may influence the generalizability of the results. (b) The functional parameters of the barrier, such as paracellular permeability and gut microbiota assessment, as well as the systemic and tissue bioavailability of quercetin, were outside of this scope. (c) Different sample sizes were used in analyses, as predefined subsamples were allocated for specific tests. An a priori power analysis was not carried out, which may have influenced the statistical power of some results, highlighting that future studies should include formal power calculations to strengthen the robustness and reproducibility of the findings. (d) The results are based on transcriptional analysis and should be interpreted with caution, highlighting the importance of evaluating potential inflammatory and molecular signaling pathways involved, as well as protein validation (post-transcriptional regulation and protein–protein interaction), parameters that remain as mechanistic hypotheses and need to be explored in future work.

## 5. Conclusions

Neonatal quercetin administration partially attenuated intestinal dysfunction induced by experimental cerebral palsy in a segment-dependent manner. Its effects were more evident in the jejunum, where quercetin reduced oxidative damage and modulated antioxidant defenses, while also enhancing the expression of genes related to epithelial barrier integrity, such as occludin and zonulin. Although quercetin did not fully reverse CP-induced morphological alterations, these findings indicate that early-life quercetin exposure contributes to the preservation of intestinal redox balance and epithelial barrier components following neonatal neurological injury. Therefore, quercetin may represent a promising adjuvant strategy for mitigating secondary intestinal impairments associated with cerebral palsy, reinforcing the relevance of gut–brain axis modulation in early-life interventions.

## Figures and Tables

**Table 1:** Sequences of primers used for gene expression analysis by RT-qPCR in Wistar rats with or without CP, with or without administration of quercetin, at 33 days of life.

GENE	Access Number	Forward Primer (5′-3′)	Reverse Primer (5′-3′)
*EEF1A1*	NM_175838.2	TGAACCATCCAGGCAAATC	GCATGCTATGTGGGCTGTGT
*Occludin*	NM_031329.3	TGACCTGTCTTGGGTTCTGT	TGCTGTGTATGAACACTATCCC
*Zonulin*	NM_001281379.1	GACATCACCCCCACCCTA	CCGATATCCACCACGGAGT
*MUC2*	NM_022174.1	TGGTGAATGACCCGTCCAAG	CAGGGAAAGGTCCTGGTGTC

*EEF1A1*: elongation factor 1-alpha 1; *MUC2*: mucin 2.

## Data Availability

The original contributions presented in this study are included in the article/Supplementary Material. Further inquiries can be directed to the corresponding author.

## Supplementary Materials

The following supporting information can be downloaded at https://www.mdpi.com/article/10.3390/antiox15040495/s1, Figure S1: Histomorphometry of jejunum at P33 in healthy animals or animals with CP, receiving or not receiving treatment with quercetin (10 mg/kg), intraperitoneally, from P2 to P22 (n = 7); Figure S2: Histomorphometry of ileum at P33 in healthy animals or animals with PC, receiving or not treatment with quercetin (10 mg/kg), intraperitoneally, from P2 to P22 (n = 7).

## Abbreviations

The following abbreviations are used in this manuscript:

CP	Cerebral palsy
Akt	Activated protein kinase B
AMP	Adenosine monophosphate
AMPK	AMP-activated protein kinase
DMSO	Dimethyl sulfoxide
HE	Hematoxylin and eosin
MDA	Malonaldehyde
MLCK	Myosin light-chain kinase
MUC 2	Mucin 2
NF-Κβ	Nuclear factor kappa β
Nrf2	Nuclear factor erythroid 2-related factor 2
PI3K	Phosphoinositide 3-kinase
Q	Quercetin
TBA	Thiobarbituric acid
TLR4	Toll-like receptor 4
V	Vehicle solution
