# Supplementary material for: Neonatal Quercetin Reduces Intestinal Oxidative Damage and Upregulates Tight Junction-Related Genes in a Mouse Experimental Model of Cerebral Palsy
Source: Antioxidants (Basel). 2026 Apr 16;15(4):495. doi: 10.3390/antiox15040495 (PMC13113978; doi:10.3390/antiox15040495)
Supplement: Supplementary file 1 [file antioxidants-15-00495-s001.zip › antioxidants-4209310-supplementary.pdf]

ARTICLE

NEONATAL EXPOSURE TO QUERCETIN REDUCES BIOMARKERS OF OXIDATIVE DAMAGE IN THE INTESTINE AND INCREASES THE EXPRESSION OF TIGHT JUNCTION PROTEINS IN AN EXPERIMENTAL MODEL OF CEREBRAL PALSY

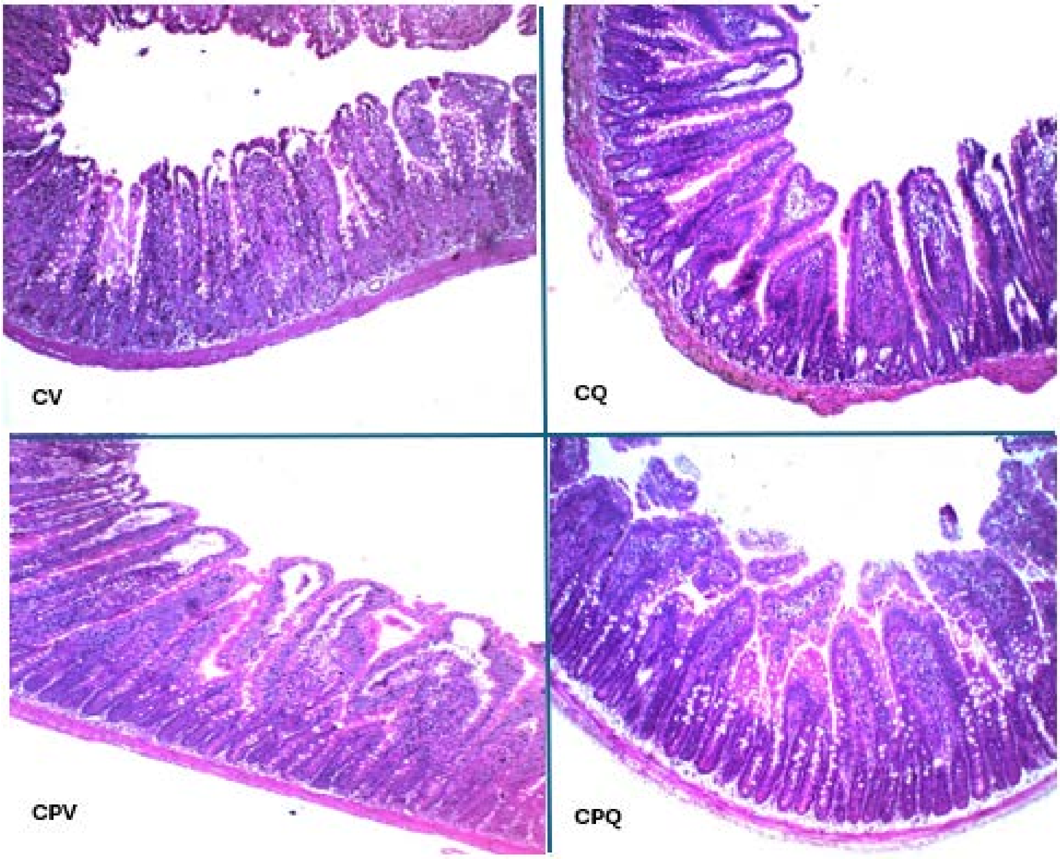

Figure S1: Histomorphometry of jejunum at P33 in healthy or with CP animals, receiving or not treatment with quercetin (10 mg/kg) intraperitoneally, from P2 to P22 (n=7). CV: Control + Vehicle, CQ: Control + Quercetin, CPV: Cerebral Palsy + Vehicle, CPQ: Cerebral Palsy + Quercetin. Representative images (40×); scale bar = 100 µm.

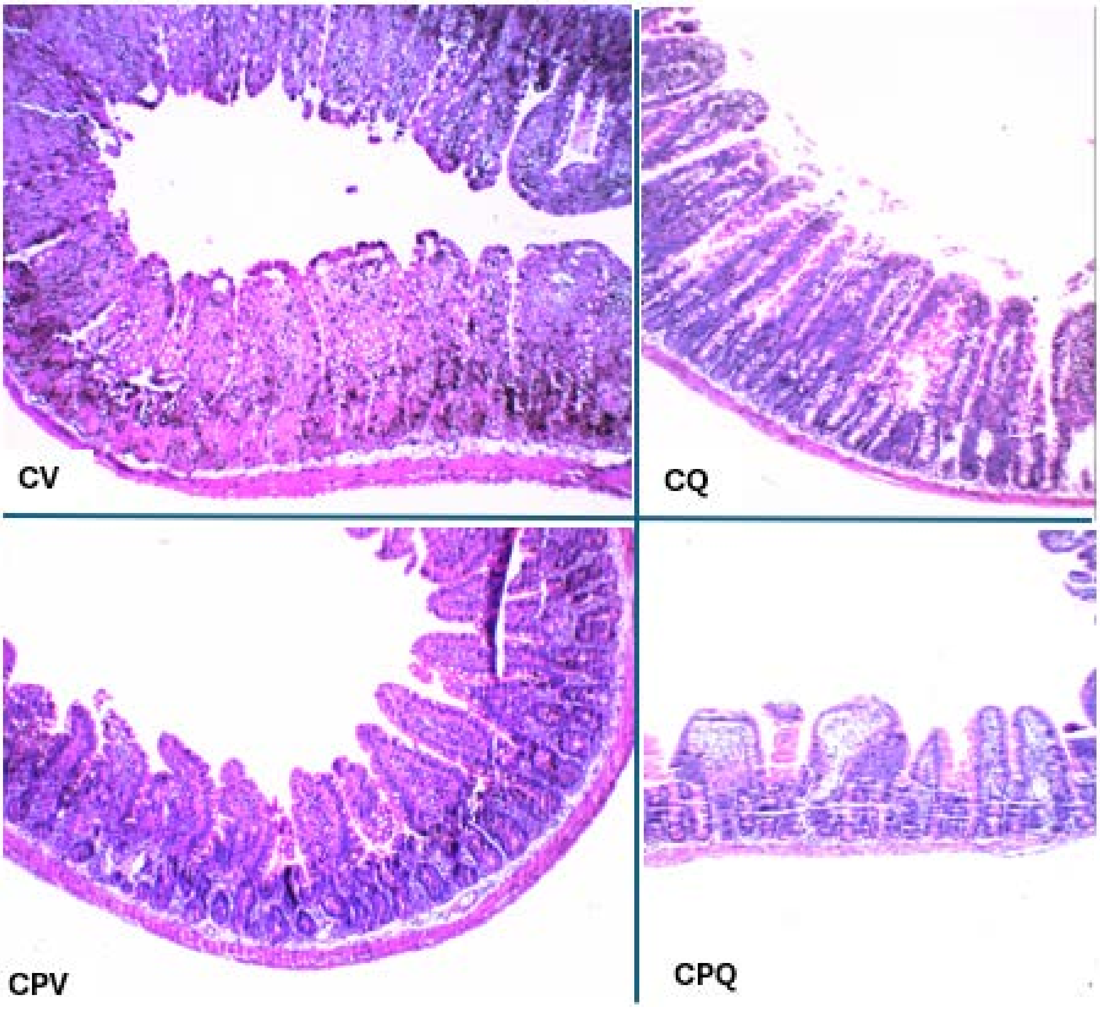

Figure S2: Histomorphometry of ileum at P33 in animals healthy or with PC, receiving or not treatment with quercetin (10 mg/kg) intraperitoneally, from P2 to P22 (n=7). CV: Control + Vehicle, CQ: Control + Quercetin, CPV: Cerebral Palsy + Vehicle, CPQ: Cerebral Palsy + Quercetin. Representative images (40×); scale bar = 100 µm.
